# Clinical characteristics of culture-negative periprosthetic joint infections: findings from an international periprosthetic joint infection registry

**DOI:** 10.5194/jbji-10-553-2025

**Published:** 2025-12-08

**Authors:** Graham S. Goh, Elise R. Naufal, Michelle M. Dowsey, Sina Babazadeh, Jesse E. Otero, Carlos A. Higuera-Rueda, Marjan Wouthuyzen-Bakker, Orthopaedic Device Infection Network (ODIN)

**Affiliations:** 1 Department of Orthopaedic Surgery, Boston University Medical Center, Boston, Massachusetts, USA; 2 Department of Surgery, St Vincent's Hospital, Melbourne, Victoria, Australia; 3 Levitetz Department of Orthopaedic Surgery, Cleveland Clinic Florida, Weston, Florida, USA; 4 OrthoCarolina Hip and Knee Center, Charlotte, North Carolina, USA; 5 Department of Medical Microbiology and Infection Prevention, University Medical Center Groningen, Groningen, the Netherlands; ➕ A full list of authors appears at the end of the paper

## Abstract

**Background**: Culture-negative periprosthetic joint infections (CN-PJIs) remain a major problem in the field of orthopedic infections. The clinical features of CN-PJI and its risk factors remain poorly defined. The purpose of this study was to elucidate the characteristics of CN-PJI. **Methods**: This was a retrospective multi-center cohort study as part of the Orthopaedic Device Infection Network (ODIN). Using real-world data from five institutions across Australia, the Netherlands and the USA, 563 cases of PJI (470 culture positive, 93 culture negative) were queried between 1995 and 2021. Patients with CN-PJI had negative cultures on pre-operative aspiration, blood or intra-operative cultures. Demographics, history of surgery on the infected joint, presenting symptoms, operative details, laboratory values and intra-operative findings were recorded. Multivariable regression was used to determine the association between these variables and culture negativity. **Results**: The prevalence of CN-PJI was 16.5 %. Bivariate analysis revealed that patients with CN-PJI were more likely to be female, have a revision arthroplasty or prior PJI, have a longer duration of symptoms and were less likely to present with fever, wound dehiscence or wound necrosis; they also had lower hemoglobin and serum CRP ( p < 0.05 for all). Using multivariable regression, the only factor significantly associated with CN-PJI was a duration of symptoms of > 12 weeks (OR 2.24, 95 % CI 1.008–4.964, p = 0.048 ). **Conclusions**: Patients with prolonged symptoms were twice as likely to have negative cultures, supporting the traditional belief that CN-PJI presents more insidiously. These clinical data should be used to guide the selection of advanced investigations.

## 1 Introduction

Periprosthetic joint infection (PJI) remains one of the most severe complications following total joint arthroplasty (TJA), adversely affecting a patient's quality of life and overall life expectancy (Mundi et al., 2024). With the rapid rise in the global demand for TJA, the burden of PJI has put tremendous pressure on healthcare systems to mitigate its downstream financial implications (Premkumar et al., 2020).

A prompt and accurate diagnosis of PJI, including pathogen identification, remains paramount to ensure optimal antimicrobial treatment of this disastrous complication. While culture remains the gold standard for diagnosing infection, a major drawback of culture-based methods is the challenge of cultivating certain micro-organisms, especially fastidious and anaerobic species, using conventional microbiological techniques (Lagier et al., 2018). This has resulted in a diagnostic conundrum known as culture-negative PJI (CN-PJI; Goh and Parvizi, 2022), which has been associated with poorer outcomes in some studies (Hersh et al., 2019; Mortazavi et al., 2011). Notwithstanding, advancements in sequencing technology have enhanced the sensitivity of traditional culture in the setting of diseases with low culture yields, including but not limited to PJI (Kullar et al., 2023). Indeed, a recent multi-center study on 301 patients with PJI found that a pathogen could be identified using next-generation sequencing (NGS) in 56 of 85 (65.9 %) culture-negative cases (Goswami et al., 2022), while another study found that additional pathogens were detected in 43 of 98 (43.9 %) cases of PJI (Thoendel et al., 2018), of which 21 had no positive cultures.

Nonetheless, one major barrier to widespread acceptance of any new technology in medicine remains the high costs, prompting some authors to suggest that these newer sequencing technologies be applied only to patients with a high clinical likelihood of CN-PJI (Abdel et al., 2019; Corona et al., 2019). However, the clinical presentation of CN-PJI remains highly heterogenous in the literature (Browning et al., 2022; Choi et al., 2013), and consistent characteristics as well as risk factors remain largely undefined. To guide the selection of appropriate investigations and, ultimately, the targeted treatment of these complex infections, additional supportive clinical data are therefore necessary.

The purpose of this multi-center study was to elucidate the clinical characteristics of CN-PJI. We present novel real-world data utilizing an international PJI registry to substantiate these findings. Our hypothesis was that the clinical characteristics of CN-PJI would be more insidious compared to that of culture-positive PJI (CP-PJI).

## 2 Methods

### 2.1 Patient cohort

This was a retrospective multi-center cohort study as part of the Orthopaedic Device Infection Network (ODIN; Naufal et al., 2024), an international collaborative involving five institutions from Australia, the Netherlands and the USA. All institutions were large tertiary care centers in the respective countries. Between 1995 and 2021, 563 cases of PJI were queried from the ODIN database. Patients were included in this international database if they met the MSIS 2011 (Parvizi et al., 2011), ICM 2018 (Parvizi et al., 2018) or EBJIS 2021 (McNally et al., 2021) criteria for PJI of a total hip or knee arthroplasty and subsequently underwent surgical treatment for PJI. Patients with septic arthritis or osteomyelitis, fracture-related infections, PJI of shoulder arthroplasties and PJI that did not undergo surgery were excluded from the ODIN registry. Patients were categorized as CN-PJI if they had negative cultures on pre-operative aspiration and intra-operative cultures. In total, the cohort comprised 470 cases of CP-PJI (83.5 %) and 93 cases of CN-PJI (16.5 %).

### 2.2 Database maintenance and data collection

The ODIN employs a strategy of distributed data collection coupled with centralized data analysis. Each database was maintained by a dedicated research coordinator at each respective institution. Standardized data collection was facilitated through the creation of a unified case entry form and a data dictionary that was housed locally. Through an iterative process, this form was developed to include information on each patient's demographics, operative details of their index arthroplasty, history of surgery on the affected joint, clinical presentation for suspected infection, pre-operative diagnostics and intra-operative findings. Prior antibiotic usage was not collected. Missing data accounted for < 20 % of each variable collected.

### 2.3 Statistical analysis

All descriptive statistics were presented as means and standard deviation for normally distributed data as determined by Kolmogorov–Smirnov testing unless otherwise specified. Preliminary bivariate analyses using independent t tests for continuous variables and chi-squared tests for categorical variables were performed to identify significant patient and surgical factors that were associated with CN-PJI. Variables with a p value of < 0.2 on bivariate analyses were incorporated into the multivariable logistic regression model in order to determine the independent association between these variables and culture negativity. All statistical tests were performed using Stata software, version 12.0 (Stata Corp., College Station, TX). Statistical significance was defined as a p value of < 0.05.

## 3 Results

In the cohort of 563 patients, there were 470 cases of CP-PJI (83.5 %) and 93 cases of CN-PJI (16.5 %). Mean age was 66.2 years, and 51 % of patients were female. Bivariate analysis revealed that patients with CN-PJI were more likely to be female, infections of revision TJA instead of primary TJA, had recurrent PJIs (i.e., more than one PJI event) and had chronic PJIs (Table [Table T1]).

**Table 1 T1:** Clinical characteristics of the cohort ( n = 563 ). Bold values indicate statistical significance ( p<0.05 ).

	Culture negative ( n = 93 )	Culture positive ( n = 470 )	p value
Age (years)	67 ± 13	66 ± 13	0.29
Sex			**< 0.001**
Female	36 (56 %)	234 (50 %)	
Male	52 (39 %)	236 (50 %)	
BMI (kg m^−2^)	32 ± 9	34 ± 9	0.65
CCI	3 ± 2	4 ± 2	0.12
Joint			0.26
Hip	56 (60 %)	250 (53 %)	
Knee	37 (40 %)	220 (47 %)	
Surgery type			**< 0.001**
Primary	50 (54 %)	291 (62 %)	
Revision	37 (40 %)	179 (38 %)	
Endoprosthesis	4 (4 %)	45 (10 %)	0.25
First-time infection	71 (76 %)	395 (84 %)	**< 0.001**
Infection onset			**< 0.001**
Early ( < 3 months)	27 (29 %)	189 (40 %)	
Chronic ( ≥ 3 months)	45 (48 %)	203 (43 %)	
Late-acute (hematogenous)	2 (2 %)	64 (14 %)	
Duration of symptoms			**< 0.001**
< 4 weeks	20 (22 %)	253 (54 %)	
4–12 weeks	19 (20 %)	79 (17 %)	
> 12 weeks	33 (35 %)	126 (27 %)	
Clinical presentation			
Fever	7 (8 %)	90 (19 %)	**< 0.001**
Wound dehiscence	8 (9 %)	65 (14 %)	**< 0.001**
Wound necrosis	1 (1 %)	12 (3 %)	**< 0.001**
Pre-operative labs			
Hemoglobin (mmol L^−1^)	34 ± 39	54 ± 44	**< 0.001**
Creatinine ( µmol L^−1^)	180 ± 831	228 ± 475	0.51
ESR (mm h^−1^)	42 ± 33	49 ± 34	0.12
CRP (mg L^−1^)	122 ± 390	284 ± 669	**0.041**

Values given as mean ± SD or N (%). BMI, body mass index; CCI, Charlson comorbidity index; ESR, erythrocyte sedimentation rate; CRP, C-reactive protein.

In addition, patients with CN-PJI had a longer duration of symptoms and were less likely to present with fever, wound dehiscence or wound necrosis. Their pre-operative hemoglobin and CRP levels were also significantly lower ( p < 0.05 for all). In contrast, age, body mass index, Charlson comorbidity index, baseline creatinine levels and pre-operative erythrocyte sedimentation rate (ESR) were not significant factors.

Using multivariable regression to control for possible confounders, the only factor significantly associated with CN-PJI was a duration of symptoms of > 12 weeks (OR 2.23, 95 % CI 1.005–4.934, p = 0.048 ; Table [Table T2]). Other variables in the regression analysis such as sex, Charlson comorbidity index (CCI), joint (hip versus knee), infection onset, presence of fever, hemoglobin, pre-operative erythrocyte sedimentation rate and C-reactive protein levels were not significant factors.

**Table 2 T2:** Multivariable analysis for factors associated with negative cultures. Bold values indicate statistical significance ( p<0.05 ).

Variable	Adjusted odds ratio	95 % lower	95 % upper	p value
Sex				
Male	ref			
Female	1.506	0.902	2.515	0.118
CCI	0.944	0.831	1.072	0.375
Joint				
Hip	ref			
Knee	1.309	0.805	2.126	0.277
Endoprosthesis	0.480	0.168	1.368	0.169
Infection onset				
Early ( < 3 months)	ref			
Chronic ( ≥ 3 months)	0.890	0.474	1.672	0.718
Late-acute (hematogenous)	0.495	0.134	1.833	0.291
Fever	0.758	0.347	1.654	0.486
Duration of symptoms				
< 4 weeks	ref			
4–12 weeks	2.416	0.982	5.942	0.055
> 12 weeks	2.227	1.005	4.934	**0.049**
Hemoglobin	0.994	0.987	1.002	0.140
ESR	0.999	0.990	1.009	0.908
CRP	1.000	0.999	1.001	0.677

CCI, Charlson comorbidity index; ESR, erythrocyte sedimentation rate; CRP, C-reactive protein.

## 4 Discussion

This large multi-center registry study sought to elucidate the clinical characteristics of CN-PJI, a diagnostic entity that continues to challenge orthopedic surgeons around the world. Our findings highlight the fact that CN-PJI represents a distinct clinical subgroup, with a longer symptom duration as the sole independent predictor after controlling for confounding factors. These findings build on prior literature, suggesting that CN-PJI often manifests in a more indolent fashion compared to CP-PJI, underscoring the importance of clinical suspicion even in the absence of classic infectious signs.

The prevalence of CN-PJI in our study was 16.5 %, which aligns with prior reports estimating rates of 7 %–42 % as well as one systematic review demonstrating a pooled rate of 11 % based on a random-effects model (Berbari et al., 2007; Goh and Parvizi, 2022; Reisener and Perka, 2018). This variability highlights both the limitations of current microbiological diagnostics and the lack of standardized thresholds for what constitutes “culture negative”. Differences in sampling technique, culture transport and laboratory techniques could also account for the wide range of incidence rates reported in the literature. Furthermore, the relatively high rate in our cohort likely reflects the inclusion of chronic and revision cases – scenarios where antibiotic exposure or slow-growing pathogens are more prevalent (Tande and Patel, 2014; Thoendel et al., 2018). Bivariate analysis revealed several features suggestive of a more insidious presentation in CN-PJI, including lower systemic inflammatory markers, the absence of local wound complications and a greater proportion of chronic cases. These findings are consistent with previous observations that CN-PJI lacks the overt clinical signs seen in CP-PJI and may be driven by slow-growing or fastidious organisms (Berbari et al., 2007; Choi et al., 2013). For instance, Browning et al. (2022) found that patients with CN-PJI had a lower mean C-reactive protein and were more likely to be female compared to CP-PJI patients, while Choi et al. (2013) found that these patients had a lower mean ESR and were more likely to have undergone revision arthroplasty. After applying multivariable regression, the only factor that remained significant was a symptom duration of greater than 12 weeks. This underscores the clinical assertion that prolonged joint symptoms – especially in the absence of systemic signs – should raise concern for CN-PJI, even in the face of negative cultures. Notwithstanding, caution should be taken when interpreting this finding as prior antimicrobial exposure is also an important factor that was not investigated in the current study; in particular, the use of antimicrobial therapy during the 3 months prior to the diagnosis of CN-PJI has been shown to be present in 53 % of culture-negative cases (Berbari et al., 2007).

The diagnostic limitations of conventional culture have prompted a rise in interest in molecular techniques such as next-generation sequencing (NGS), which has demonstrated improved pathogen detection in CN-PJI (Tarabichi et al., 2018a). Metagenomic next-generation sequencing (MG-NGS), a high-throughput sequencing technique (Tarabichi et al., 2018a), enables complete bacterial genomes as well as their resistance genes to be sequenced (Ruppé et al., 2017). An improved sensitivity compared to traditional culture has been reported in multiple studies (Huang et al., 2019b; Ruppé et al., 2017; Street et al., 2017; Tarabichi et al., 2018b, a; Thoendel et al., 2018), with some demonstrating the ability of NGS to isolate the infective organism(s) in up to 82 % of culture-negative cases (Tarabichi et al., 2018a). Another newer technique is metatranscriptomic NGS (MT-NGS), which, unlike MG-NGS, is able to analyze gene expression and distinguish between active and inactive genetic pathways (Huang et al., 2019a). Multiplex polymerase chain reaction (PCR) assay is another option that can detect the most clinically relevant pathogens and resistance genes, including as mecA- and carbapenemase-encoding genes, within 1 h of sampling (Jeong et al., 2025). The aforementioned molecular methods not only circumvent the technical difficulties associated with culture methods (e.g., prolonged transport time, insufficient incubation time) but also enable pathogen identification despite prior antimicrobial therapy without compromised sensitivity (Cazanave et al., 2013; Fang et al., 2018). However, the high cost and lack of accessibility remain major barriers to widespread implementation. In line with prior recommendations from the 2018 International Consensus Meeting (ICM) that advocate for the judicious deployment of advanced diagnostics based on pre-test probability (Abdel et al., 2019; Corona et al., 2019), the present findings support the targeted application of molecular diagnostics or extended culture protocols in patients with prolonged unexplained joint symptoms, in addition to patients who may have inadvertently received antimicrobials prior to culture sampling.

The clinical implications of this study are twofold. First, CN-PJI should not be viewed as a diagnostic failure but possibly as a different biological phenotype of infection – one potentially driven by host–pathogen dynamics that evade traditional detection methods. Second, the recognition of prolonged symptomatology as a distinguishing feature may guide the appropriate use of adjunctive diagnostics such as NGS, changes in empiric antimicrobial selection and closer clinical follow-up.

This study had several limitations. As a registry-based analysis, data heterogeneity and missing variables such as prior antibiotic exposure, organism-specific testing, specimen culture and transport methodologies (e.g., transport media, incubation duration), and detailed operative factors could not be accounted for. Since prior antibiotic exposure is one of the strongest predictors of culture negativity, the absence of this variable could have confounded the analysis, and hence a symptom duration of > 12 weeks should not be overinterpreted as the sole predictor of culture-negative infections. Given the international nature of this multi-center registry, one of three different diagnostic criteria (MSIS, 2011; ICM, 2018; EBJIS, 2021) were used to classify patients with PJI and include them in the study, which could have affected the classification of CN-PJI. The relatively low number of CN-PJI cases limited the statistical power to detect weaker associations. Another limitation of this study is the differential missingness observed for several baseline variables, particularly infection onset and symptom duration, which were more pronounced in the culture-negative cohort. In these cases, incomplete documentation often reflected real-world clinical factors such as outside referrals, prior antibiotic exposure or indolent symptom evolution, leading to missingness that was structural and likely not random. Because these variables lacked the consistent clinical anchors required for valid statistical reconstruction, approaches such as imputation or formal sensitivity analyses were not feasible and could have introduced additional bias. As a result, we elected to report all available data transparently and acknowledge that the imbalance in missingness may contribute to residual confounding that should be considered when interpreting our findings. Outcome data (e.g., reinfection rates) were not available, and it remains uncertain if CN-PJI is truly a different biological phenotype. Lastly, while our findings are hypothesis generating, association does not imply causation, and they require validation in prospective studies with standardized diagnostic protocols and the incorporation of molecular techniques.

In conclusion, utilizing real-world data from an international PJI registry, this study found that CN-PJI is a clinically distinct subset of PJI, marked by a longer, more indolent disease course. A symptom duration exceeding 12 weeks may serve as a key clinical marker in identifying patients who would benefit from advanced microbiological testing or empiric treatment strategies. Improved characterization of this subgroup is crucial for tailoring diagnostic and therapeutic approaches in an era of precision orthopedics.

## Data Availability

Publicly accessible data are not available, as they belong to the Orthopaedic Device Infection Network (ODIN), an international collaboration addressing peri-prosthetic joint infections of knee and hip arthroplasty.

## Code availability

Publicly accessible software code is not available, but can be obtained from the corresponding author upon reasonable request.

## Team list

The Orthopaedic Device Infection Network (ODIN): Simon W. Young (Department of Orthopaedic Surgery, North Shore Hospital, Auckland, Aotearoa / New Zealand; Department of Surgery, Faculty of Medical and Health Sciences, University of Auckland, Auckland, Aotearoa / New Zealand), Alex Soriano (Department of Infectious Diseases, Hospital Clínic of Barcelona, Barcelona, Spain), Jarrad Stevens (Department of Orthopaedic Surgery, St. Vincent's Hospital Melbourne, Melbourne, Victoria, Australia), Craig Aboltins (Department of Infectious Diseases, Northern Health, Epping, Victoria, Australia; Department of Medicine, Northern Health, The University of Melbourne, Melbourne, Victoria, Australia) and Cade Shadbolt (Department of Surgery, St Vincent's Hospital Melbourne, Melbourne, Victoria, Australia).
